# Optimizing Chronic Migraine Care: The Impact of Advanced Practice Nurse Involvement in OnabotulinumtoxinA Administration

**DOI:** 10.31083/RN45618

**Published:** 2026-03-10

**Authors:** Eulalia GINE-CIPRES, Marta TORRES-FERRUS, Víctor J GALLARDO, Alicia ALPUENTE RUIZ, Edoardo CARONNA, Laura GOMEZ-DABO, Patricia POZO-ROSICH

**Affiliations:** ^1^Headache Unit, Neurology Department, Vall d'Hebron University Hospital, 08035 Barcelona, Spain; ^2^Headache Research Group, Vall d'Hebron Research Institute, Autònoma University of Barcelona, 08035 Barcelona, Spain

**Keywords:** migraine, botulinum toxin type A, treatment, headache, advanced practice nursing

## Abstract

**Background:**

Chronic migraine is a disabling and prevalent neurological disease that significantly impacts patients' quality of life. OnabotulinumtoxinA (OnabotA) is a safe and effective chronic migraine preventive treatment. The objective was to evaluate adherence to the recommended time interval between consecutive OnabotA injections according to the Phase III Research Evaluating Migraine Prophylaxis Therapy (PREEMPT) protocol, and to analyze changes in the interval adherence before and after the introduction of an Advanced Practice Nurse (APN) in a specialized Headache Unit of a tertiary hospital.

**Methods:**

This was a retrospective study performed in a Spanish specialized Headache Clinic. Patients diagnosed with chronic migraine and treated with OnabotA following the PREEMPT protocol were included, before and after the introduction of APNs. Treatment was considered interval-compliant if the injection interval ranged from 75 to 105 days. The data collected included three 18-month periods. Statistical analyses examined differences across periods using R-Studio.

**Results:**

A total of 2991 participants were included, of whom 83.8% (2507/2991) were women, with a median age [interquartile range (IQR)] of 48.0 [40.0–57.0] years old. A statistically significant association between the introduction of the APN and improved adherence to the PREEMPT protocol was observed over time (Z = –19.60, *p* < 0.001). Adherence rates increased from 52.1% in P1 to 76.1% in both P2 and P3. The median time between visits decreased from 105 days in P1 to 96 days in P2, with a slight increase to 98 days in P3.

**Conclusion:**

The involvement of advanced practice nurses in OnabotA administration decreased the time interval between infiltrations and improved adherence to the PREEMPT protocol. These findings highlight the APN's role in optimizing patient care.

## 1. Introduction

Chronic migraine (CM) is a debilitating, prevalent, neurological disease that significantly impacts patients’ quality of life [1]. Migraine affects over 1 billion people worldwide and in Spain, it affects 5 million people, of which 500,000 suffer CM. For this reason, specific headache consultations are usually very overburdened [2, 3]. In this population, preventive treatment is mandatory and is key to reducing the burden in people with CM [4]. OnabotulinumtoxinA (OnabotA) is an effective and safe preventive treatment for CM reducing headache frequency, medication use, and improving quality of life as demonstrated by the PREEMPT studies which used a 155 U to 195 U fixed-dose protocol with pericranial subcutaneous injections every 12 weeks [5]. In Spain, OnabotA was approved and financed in 2012 for the preventive treatment of CM and is widely administered by neurologists specializing in headache management [6].

An Advanced Practice Nurse (APN) is a highly trained nurse with advanced clinical skills, capable of managing illnesses, providing specialized health education to society, overseeing chronic disease treatment, and continuously updated with recent technological advancements, methodologies, and healthcare practices [7]. If individuals respond to OnabotA treatment, the administration and evaluation of OnabotA is done every 12 weeks. This creates increasing demand among responders who require treatment, especially straining procedural capacity within Headache Clinics. The growing number of patients requiring a 3-month follow-up also increases the need for patient education and monitoring. An APN was recruited and incorporated into the team. This change was evaluated by comparing it to previous clinical practice, including adherence to the Phase III Research Evaluating Migraine Prophylaxis Therapy (PREEMPT) protocol schedule.

In 2019, an APN joined the Headache Unit as part of an organizational initiative to improve access to care for patients with chronic migraine. The nurse received direct training from neurologists specialized in headache medicine and was closely supervised during the initial phase until formal delegation of the OnabotA administration technique was approved. Additionally, the APN completed the Headache Specialization Course of the Catalan Society of Neurology, ensuring the necessary theoretical and practical competence for performing the procedure.

Hence, the objective was to evaluate adherence to the recommended time interval between consecutive OnabotA injections according to the PREEMPT protocol, and to analyze changes in the interval adherence before and after the introduction of an APN in a specialized Headache Unit of a tertiary hospital.

## 2. Methods

This is a retrospective study performed in a Spanish specialized Headache Clinic.

We included adult patients attended at the Headache Outpatient Clinic diagnosed with CM and treated with OnabotA following the PREEMPT protocol. For the outlined objective, the variables extracted from the electronic medical record system were the patient record number, age, sex, visit dates of each OnabotA administration, and the healthcare professional (neurologist or APN) who performed the procedure. Treatment was considered interval-compliant with the PREEMPT protocol if the interval between injections ranged from 75 to 105 days. Although the PREEMPT protocol recommends administration every 12 weeks, in clinical practice these intervals is operationalized as approximately three months (90 days). To account for real-world scheduling variability, a ±15-days margin was applied, resulting in the 75–105-days interval adherence window used in this study. Each injection administered during the study period was considered as an individual data point for adherence analysis. Consequently, the number of administrations may exceed the total number of patients, as some patients received more than one treatment during the same period. The data collection included three 18-month periods: the first (P1), from 01/06/2018 to 31/12/2019, with OnabotA administered exclusively by neurologists; the second (P2), from 01/03/2021 to 30/09/2022; and the third (P3), from 01/03/2023 to 30/09/2024. Both periods’ infiltrations were conducted by neurologists and the APN.

Statistical analysis was conducted using R-Studio v4.1.2 (The R Foundation for Statistical Computing, Vienna, Austria; https://www.r-project.org). Nominal variables (sex, healthcare professional, and time-intervals) were presented as frequencies (percentages), while median and interquartile range (IQR) were reported for quantitative variables, including age and waiting time (in days) between OnabotA visits. Normality of quantitative variables was confirmed through Q-Q plots and the Shapiro-Wilk test. Differences between healthcare professionals (neurologist vs. APN) were evaluated using an unpaired *t*-test, and the linear-by-linear association test was used to examine trends across study periods for categorical variables. Effect sizes for ordered associations were calculated using Kendall’s tau-b with 95% confidence intervals. A 5% significance level was used for all tests.

## 3. Results 

**Fig. 1.**
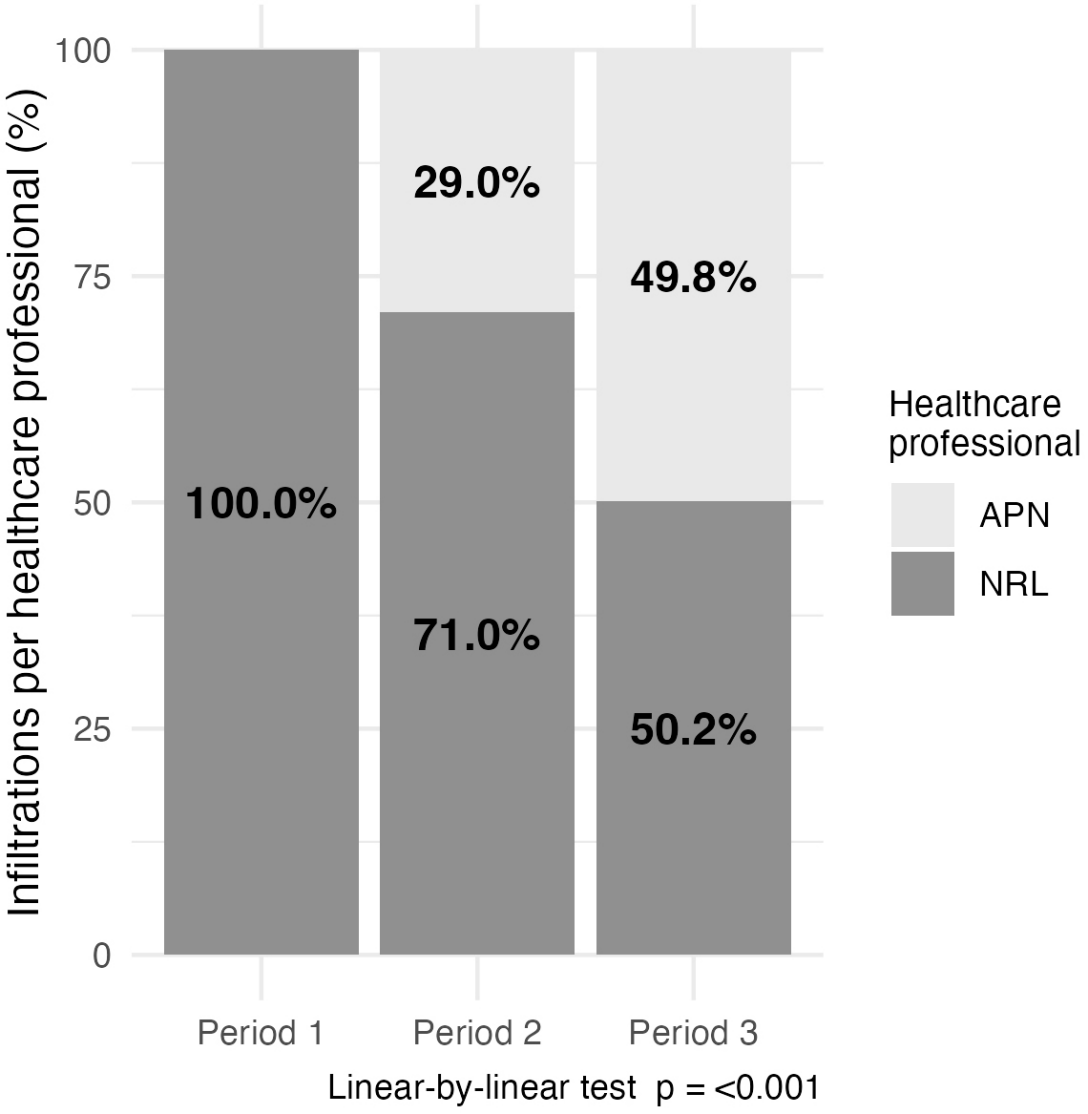
**Infiltrations per healthcare professional (%)**. APN, advanced practice nurse; NRL, neurologist.

A total of 2991 participants were included, of whom 83.8% (2507/2991) were women, with a median age [IQR] of 48.0 [40.0–57.0] years old. During P1, 2718 visits were recorded, with all infiltrations performed exclusively by neurologists. In P2, 4267 administrations were documented, with 71.0% (3031/4267) of infiltrations conducted by neurologists and 29.0% (1236/4267) by APNs. During P3, 3826 administrations were recorded, with infiltrations nearly evenly split between neurologists (50.2%; 1921/3826) and APNs (49.8%; 1905/3826) (Fig. 1).

**Fig. 2.**
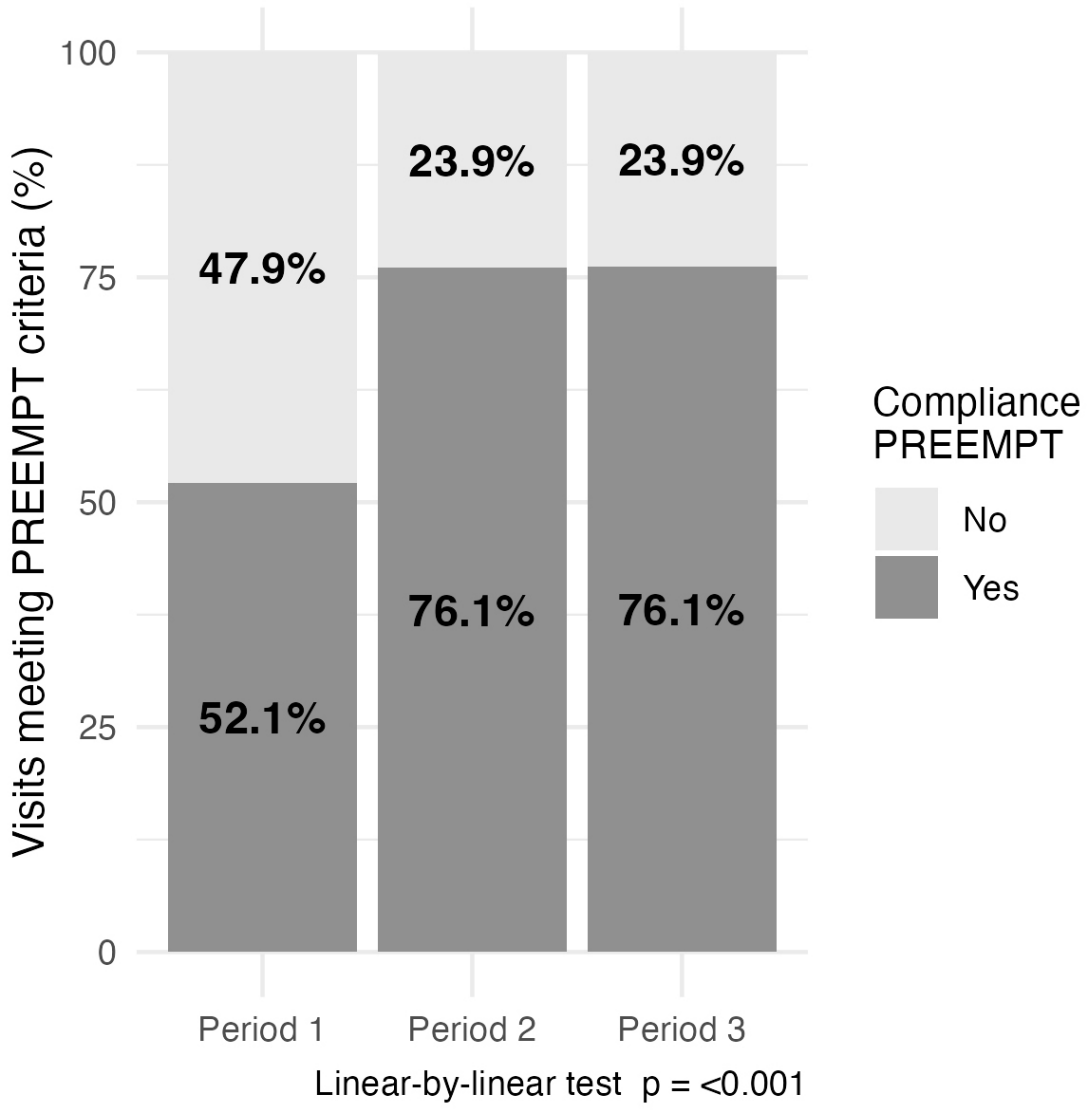
**Visits meeting Phase III Research Evaluating Migraine Prophylaxis Therapy (PREEMPT) criteria (%)**.

Adherence to the PREEMPT protocol showed a statistically significant association with the study period (Z = –19.60, *p* < 0.001), with an effect size of τb = 0.17 (95% CI [0.15, 0.19]) (Fig. 2). In P1, 52.1% (1417/2718) of administrations adhered to the interval of time-compliance PREEMPT protocol, while adherence during P2 was 76.1% (3246/4267), and 76.1% (2913/3826) during P3, significant decrease in administration time-intervals was observed across periods. The median time between visits decreased from P1 to P3, accompanied by a narrowing of the time interval, indicating more consistent and shorter visit durations in P2 and P3 (P1: 105.0 [93.0–116.0] days, P2: 96.0 [90.0–105.0] days, P3: 98.0 [91.0–105.0]), with a median reduction of –9 days from P1 to P2 and a slight increase of +2 days from P2 to P3.

## 4. Discussion

The administration of OnabotA by headache nurses is a key component of APN professional practice [8]. In this study, the involvement of APNs in the administration of OnabotA was associated with shorter injection intervals and improved adherence to the PREEMPT protocol, which is critical for the effective management of CM [9]. Adherence to the PREEMPT protocol directly benefits individuals with CM by improving the quality of care [9], while for the medical team, the presence of the APNs reduces outpatient care pressure by reducing the number of visits exclusively managed by neurologists. It promotes interdisciplinary collaboration. We believe that improved adherence to the recommended OnabotA administration interval directly enhances quality of care because consistent 12-week dosing minimizes symptom fluctuation and ensures more stable migraine control, while the increased scheduling efficiency provided by the APN reduces waiting times and reinforces a patient-centred care pathway [10]. This improvement may be partially attributed to an increase in human resources allocated to treatment administration. However, other aspects, such as enhanced communication between patients and APNs, greater access to the Headache Clinic, and management of agendas, may have also contributed to the improved adherence observed. Similar European experiences have described the integration of nurse-led OnabotA administration as a safe and efficient model of care within multidisciplinary headache services, improving access and treatment continuity for patients with chronic migraine [8].

The slight increase of +2 days observed in P3 suggests that the system may be reaching its capacity with one nurse, potentially indicating a saturation of available resources. Although the total number of OnabotA administrations decreased in the last period, adherence rates remained stable. Therefore, this variation does not seem to be related to the introduction or availability of other preventive treatments, but rather to internal organizational adjustments in appointment scheduling and patient flow. This trend underscores the need for further reinforcement of APN with another nurse to maintain optimal adherence rates and ensure continued accessibility to treatment. Expanding the involvement of APNs could help sustain the improvements observed in P2 while preventing potential delays in treatment administration as patient demand continues to grow.

It is important to note that, in clinical practice, some well-controlled patients may tolerate slightly extended treatment intervals without loss of efficacy [11]. Although this study focused on adherence to the PREEMPT-recommended 12-week schedule rather than clinical outcomes, this aspect could be relevant when interpreting adherence data. Future studies could explore whether individual variability in response allows flexibility in injection timing without compromising treatment effectiveness.

A limitation of this study is that adherence was used as a surrogate outcome, without including direct clinical efficacy measures such as headache frequency, acute medication use, or patient-reported outcomes. Therefore, although we could not objectively confirm clinical improvement in our cohort, prior evidence suggests that maintaining regular 12-week dosing is associated with positive migraine control [12]. These data were not available within the retrospective design; however, future prospective studies within our group are already addressing these outcomes, as well as evaluating the tolerability of OnabotA administration, to further assess the clinical impact of improved adherence. A further limitation is that the study was conducted in a single tertiary headache unit, which may restrict the generalizability of the findings to other healthcare settings or models of nurse-led care. Although the COVID-19 period was not included, post-pandemic organisational changes may still act as residual confounders. Nonetheless, a key strength of this work is the large real-world sample spanning multiple time periods, which enhances the reliability of the observed associations.

The approval and integration of the APN in Headache Clinics and care has played a pivotal role in developing this activity. This organizational change has allowed for greater scheduling flexibility, optimized appointment management, and improved continuity of care for patients requiring OnabotA administration. This is the first study in Spain to quantify the organizational impact of introducing an APN into chronic migraine management, providing real-world evidence on improved adherence to recommended injection intervals. These findings offer a valuable insight for healthcare planning and optimization of multidisciplinary headache models.

Future research could explore patients’ perspectives on adherence and perceived differences between nurse- and physician-led care, as an integrated approach to care seems to be positively impacting treatment adherence.

## 5. Conclusion

This study demonstrated that the incorporation of APNs was associated with a higher number of infiltrations, shorter administration intervals, and improved adherence to the PREEMPT protocol. These changes contributed directly to enhanced care for individuals with chronic migraine.

## Availability of Data and Materials

The data supporting the findings of this study are not publicly available due to institutional data protection and confidentiality policies. Data availability complies with the journal’s editorial policies and formats as outlined by IMR Press. The datasets are available from the corresponding author upon reasonable request.

## References

[1] (2018). Headache Classification Committee of the International Headache Society (IHS) The International Classification of Headache Disorders, 3rd edition. *Cephalalgia: an International Journal of Headache*.

[2] Ashina M, Katsarava Z, Do TP, Buse DC, Pozo-Rosich P, Özge A (2021). Migraine: epidemiology and systems of care. *Lancet (London, England)*.

[3] Steiner TJ, Stovner LJ, Katsarava Z, Lainez JM, Lampl C, Lantéri-Minet M (2014). The impact of headache in Europe: principal results of the Eurolight project. *The Journal of Headache and Pain*.

[4] Lipton RB (2024). Preventive Treatment of Migraine.

[5] Dodick DW, Turkel CC, DeGryse RE, Aurora SK, Silberstein SD, Lipton RB (2010). OnabotulinumtoxinA for treatment of chronic migraine: pooled results from the double-blind, randomized, placebo-controlled phases of the PREEMPT clinical program. *Headache*.

[6] Torres-Ferrus M, Gallardo VJ, Alpuente A, Pozo-Rosich P (2020). Influence of headache pain intensity and frequency on migraine-related disability in chronic migraine patients treated with OnabotulinumtoxinA. *The Journal of Headache and Pain*.

[7] Sevilla Guerra S, Zabalegui A, Comellas Oliva M, Estrem Cuesta M, Martín-Baranera M, Ferrús Estopà L (2022). Advanced practice nurses: Analysis of their role from a multicentre cross-sectional study. *International Nursing Review*.

[8] Rasmussen AV, Jensen RH, Gantenbein A, Sumelahti ML, Braschinsky M, Lagrata S (2024). Consensus recommendations on the role of nurses in headache care: A European e-Delphi study. *Cephalalgia: an International Journal of Headache*.

[9] Lipton RB, Rosen NL, Ailani J, DeGryse RE, Gillard PJ, Varon SF (2016). OnabotulinumtoxinA improves quality of life and reduces impact of chronic migraine over one year of treatment: Pooled results from the PREEMPT randomized clinical trial program. *Cephalalgia: an International Journal of Headache*.

[10] Ruscheweyh R, Athwal B, Gryglas-Dworak A, Frattale I, Latysheva N, Ornello R (2020). Wear-Off of OnabotulinumtoxinA Effect Over the Treatment Interval in Chronic Migraine: A Retrospective Chart Review With Analysis of Headache Diaries. *Headache*.

[11] Cernuda-Morollón E, Ramón C, Larrosa D, Alvarez R, Riesco N, Pascual J (2015). Long-term experience with onabotulinumtoxinA in the treatment of chronic migraine: What happens after one year?. *Cephalalgia: an International Journal of Headache*.

[12] Blumenfeld AM, Stark RJ, Freeman MC, Orejudos A, Manack Adams A (2018). Long-term study of the efficacy and safety of OnabotulinumtoxinA for the prevention of chronic migraine: COMPEL study. *The Journal of Headache and Pain*.

